# A novel pathogenetic factor of laryngeal attack in hereditary angioedema? Involvement of protease activated receptor 1

**DOI:** 10.1186/s13223-022-00699-7

**Published:** 2022-07-04

**Authors:** Henriette Farkas, Csilla Máj, István Kenessey, Anna Sebestyén, Ildikó Krencz, Judit Pápay, László Cervenak

**Affiliations:** 1 Hungarian Angioedema Reference Center, Department of Internal Medicine and Hematology, Semmelweis University, Budapest, Hungary; 2 Department of Internal Medicine and Hematology, Semmelweis University, Szentkirályi u. 46, Budapest, 1088 Hungary; 3 Department of Pathology, Szent György University Hospital, Székesfehérvár, Hungary; 4 2nd Department of Pathology, Semmelweis University, Budapest, Hungary; 5 1st Department of Pathology and Experimental Cancer Research, Semmelweis University, Budapest, Hungary

**Keywords:** Hereditary angioedema, C1-inhibitor deficiency, Immunohistochemistry, Laryngeal edema, Protease activated receptor 1, Bradykinin receptor

## Abstract

**Background:**

Hereditary angioedema (HAE) is a rare, life-threatening disease. The knowledge about the molecular pathogenesis of HAE has derived mainly from investigating blood samples. However, limited data are available on the role of the molecular mechanisms in the affected tissues during HAE attack.

**Objective:**

The aim of our study was to explore the histological changes occurring in HAE attacks.

**Methods:**

Post mortem macro-, microscopic and immunohistological assessment of upper airway tissues of a patient with HAE due to C1 inhibitor deficiency (C1-INH-HAE) type 2 who died from laryngeal HAE attack was compared with a non-HAE patient who died from other condition without any signs of angioedema.

**Results:**

Compared to the control patient, we demonstrated stronger T cell/monocyte infiltration and a more intense C1-INH staining in the C1-INH-HAE patient. The expression of both bradykinin receptors (B1/B2) was observed with a slightly lower level in the C1-INH-HAE patient than in the control patient. PAR1 expression was strongly reduced in the C1-INH-HAE patient suggesting overactivation of this hyperpermeability inducing receptor.

**Conclusion:**

Our unique case and novel results correspond to the knowledge about C1-INH and BDKRs observed in plasma; however, it revealed new information about the pathomechanism of HAE attack focusing on the potential involvement of PAR1 in edema formation. This observation, if it is verified by subcutaneous biopsy studies, may designate a new therapeutic target in HAE.

## Introduction

Hereditary angioedema (HAE) due to C1 esterase inhibitor (C1-INH) deficiency (C1-INH-HAE) is a rare—estimated prevalence is 1:10000 to 1:50000—, autosomal dominant disorder caused by mutation in *SERPING1* gene and belongs to the bradykinin mediated angioedemas [1]. It is clinically characterized by recurrent, nonpruritic and self-limiting angioedema (AE) episodes without wheals in the subcutaneous tissue and/or may involve the upper airway (UAE) and intestinal mucosa [2, 3]. AE involving the submucosa of the upper airways (soft palate, pharyngeal arch, uvula, tongue, larynx, hypopharynx) can cause laryngeal obstruction, which may lead to suffocation with 30 to 50% mortality of undiagnosed or inappropriately managed cases [4, 5]. Although hereditary angioedema was first described in 1888 by Osler [6], there are still many unresolved questions regarding HAE, such as what factors trigger the edematous attacks, what determines the frequency and localization of the edematous attacks, and whether the pathophysiologic processes in the blood plasma, which have been studied mostly so far, correlate with the processes in the tissues where the edema occurs.

The term edema describes a state when the blood plasma efflux into the extravascular space exceeds the draining capacity of the lymph vessels of the tissue. In angioedema, it results from the uncontrolled hyperpermeability of the endothelial cells of the capillaries caused primarily by bradykinin. Whereas the role of bradykinin, its generation, receptors and degradation are extensively studied and described, the dysfunction of endothelial cells during and preceding the edematous process is much less understood. In the case of C1-INH-HAE, the main pillar of the pathogenesis is the excessive amount of bradykinin due to the loss of control over its generating enzymes; however, it does not fully give answer to the questions: when, where, and how intensively will an edematous attack be formed in different patients? Thus, we have to consider the substantial role of endothelial cells in the hyperpermeability signal integration. Endothelial cells have a great variety of receptors for permeability controlling factors. Besides bradykinin receptors B1R and B2R, histamine receptors, toll-like receptors, endothelin receptors, cAMP and cGMP receptors and protease activated receptors (PARs) are included amongst lots of others. For example, we previously demonstrated that complement MASP-1 (mannose-binding lectin associated serine protease 1) (as well as MASP-2) is able to induce hyperpermeability in endothelial cells via PAR1 [7, 8]. This may well be such an integrative signal since MASP-1 and MASP-2 are also under the control of C1-INH. The permeability state of the endothelial cells in a given capillary, therefore, depends on the all incoming signals, and their sum intensity determines whether edema can be formed or not.

Although the pathophysiology of HAE attacks are extensively investigated assessing plasma samples, only limited data are available on the ultrastructure and molecular pathomechanism within the affected tissues. Sheffer et al. described the histopathological and ultrastructural characteristics of different tissues (including skin, jejunal tissue and larynx) obtained from patients with hereditary angioedema during HAE attacks. They observed the presence of capillary and venular dilatation as well as mononuclear cell accumulation in edematous tissues [9]. Cesoni et al. demonstrated capillary alteration in patients with C1-INH-HAE using nailfold videocapillaroscopy [10]. Complement C4 and fibrin have been detected in the non-edematous skin of C1-INH-HAE patients [11]; however, no other immunohistological assessment was performed. Especially, we do not have such data on upper airway mucosa during laryngeal HAE attack.

A C1-INH-HAE patient of ours had died from suffocation caused by a laryngeal attack, therefore, we had the possibility to carry out the subsequent autopsy and the *post mortem* examination of the larynx of this patient besides a control subject without angioedema.

The aim of our study was to analyze and compare macroscopic- and microscopic-, histological- and immunohistochemical properties of laryngeal tissues in both patients. In our expectation, exploring the histological changes occurring during HAE attacks may bring us closer to the elucidation and understanding of the pathomechanism of C1-INH-HAE.

## Methods

### Patient 1

This 36-year-old female was diagnosed with C1-INH-HAE type II at the age of 22 years, at the Hungarian Angioedema Reference Center. Her mother and son also suffered from this disease. She was educated about the clinical manifestations and course of HAE as well as supplied with the appropriate treatment (plasma-derived C1-INH [pdC1-INH] concentrate) for relieving HAE attacks. She has undergone regular follow up at our Center. She has been experiencing limb edema once or twice a year, but airway attacks have never occurred. At the last follow-up visit 0.42 g/L C1-INH antigenic level was measured (normal range: 0.15–0.3 g/L), 32% C1-INH functional activity (normal range: 70–110%), 0.02 g/L C4 (normal range: 0.15–0.55 g/L) and 119 mg/L C1q (normal range: 60–180 mg/L). One week after the last follow-up visit, progressive facial edema developed and preceded the onset of laryngeal edema. The patient dismissed these symptoms as non-serious and did not administer the medicinal product supplied for emergency use (pdC1-INH concentrate was available at her home), and died at home. Autopsy confirmed asphyxiation by laryngeal edema as the cause of death.

### Patient 2 (control subject)

The death of this 37-year-old female patient resulted from end-stage renal failure due to bilateral kidney atrophy caused by glomerular renal disease, complicated hypertension, acute heart failure, and circulatory insufficiency. Neither her family history nor clinical diagnosis showed any sign of C1-INH deficiency. Medical history was negative regarding angioedematous episodes and autopsy report did not describe any angioedematous tissues/organs.

The study protocol was approved by the institutional review board of Semmelweis University of Budapest (Reg. No. 1067-5/2018/EUIG) in accordance with the Declaration of Helsinki.

### Sample preparation—section, macro photos, and block preparation

A complete autopsy was performed within 8 h after death with particular attention to the respiratory tract. Macro photos were captured by a digital camera (Fujifilm FinePix S5800). The larynx, the epiglottis, and the upper part of the trachea was removed and further processed as follows.

A sagittal section of the whole larynx was placed in a macro-cassette. After gross sectioning of areas of interest for histological analysis and immunohistochemistry (vocal cords, false vocal cords, epiglottis, and trachea), tissue processing was performed, and after the appropriate duration of fixation in 10% neutral buffered formalin, samples were embedded in paraffin.

### H&E and immunohistochemical staining

Hematoxylin and eosin (H&E) staining and immunohistochemistry (IHC) were performed on 4 μm-thick sections of the formalin-fixed, paraffin-embedded tissue blocks. Sections were deparaffinized by xylene and rehydrated in a graded series of ethanol and water. H&E staining was made using an automated stainer (HistoCore SPECTRA ST, Leica, Wetzlar, Germany) and glass coverslipper (CV5030, Leica, Wetzlar, Germany). IHC was performed after endogenous peroxidase blocking in 0.01% periodic acid followed by 0.01% sodium borohydride and 3% hydrogen peroxide in methanol. Antigen retrieval was carried out for 30 min in a pressure cooker (10 mM citrate buffer, pH 6). The slides were incubated with the primary antibodies (summarized in Table 1) for 90 min at room temperature. Novolink Polymer (Leica Biosystems, Buffalo Grove, IL USA) was used as a secondary detection system. The IHC reactions were visualized by 3, 3′-diaminobenzidine (DAB; Aligent, Santa Clara, CA, USA) chromogen, and sections were counterstained with hematoxylin.

**Table 1:** Parameters of primary antibodies used in the study

Specificity / Dilution	Manufacturer	Catalog number
BDKRB1 polyclonal antibody, rabbit, 1:50	Abnova Corporation	PAB26133
BDKRB2 polyclonal antibody, rabbit, 1:50	Abnova Corporation	PAB26164
PAR1 Rabbit anti-Human Polyclonal (N-Terminus) Antibody, 1:1000	LifeSpan BioSciences	LS-A2583-50
PAR2 Rabbit anti-Human Polyclonal (N-Terminus) Antibody, 1:100	LifeSpan BioSciences	LS-A252-50
PAR4 Rabbit anti-Human Polyclonal (C-Terminus) Antibody, 1:100	LifeSpan BioSciences	LS-A1311-50
Rabbit anti-Human C1-Inhibitor, 1:50	In-house	–
CD3, mmAb, clone: PS-1, 1:50	BioCare	CM110AK
CD19, mmAb, clone: CD19, 1:200	BioCare	CM310A
CD11b (ITAM) rabbit Mab; clone: EPR1344; isotype: Rabbit IgG, ready-to-use	Biogenex	BG-AN546-5 M

*BDKRB1/2* bradykinin B1/B2 receptor; *PAR1/2/4* protease activated receptor 1/2/4

### Scoring system for evaluation of immunohistochemical reactions

After reviewing the H&E-stained slides, the IHC reactions were evaluated independently by two pathologists (J.P. and I.K.) and scored on a semi-quantitative scale of 0 to 3 based on the percentage of the positive cells and the staining intensity.

## Results

### Macroscopic evaluation

**Fig. 1:**
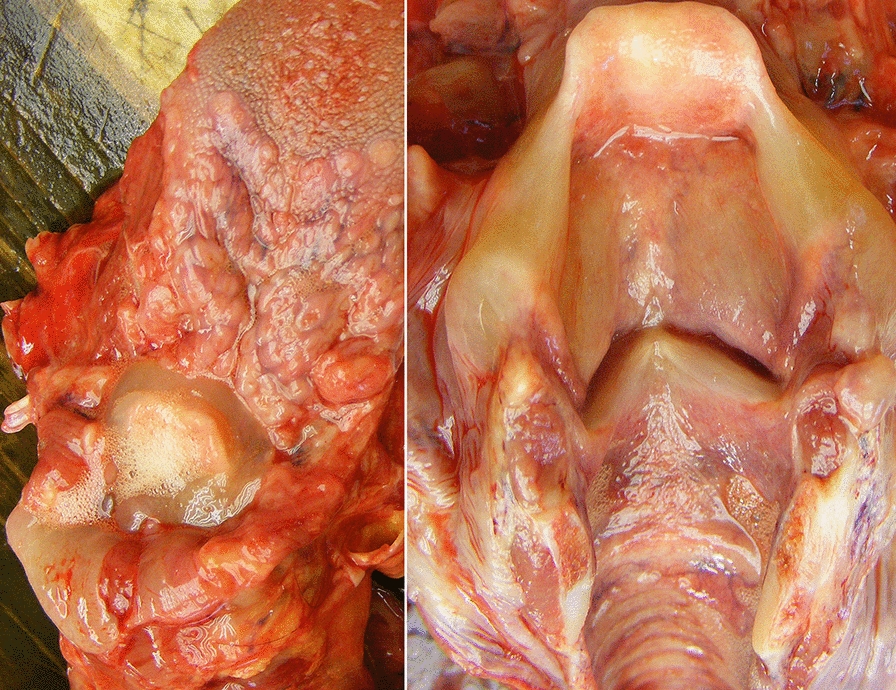
Photograph of the HAE patient’s laryngeal sample

In the HAE patient’s laryngeal sample, mucosal lining of the bronchi and trachea was non-hyperemic; it was covered by a small amount of airway secretion. The vocal cords were closed. A ball of froth was visible in the laryngeal introitus. The exploration of the larynx revealed intact vocal cords. The mucosa of the vestibular folds, the anterior and posterior aspects of the epiglottis, as well as the hypopharynx were edematous and swollen. The lumen of the esophagus was of normal size, its wall was of normal thickness, and its mucosa was intact (Fig. 1).

In the control sample, the larynx and the trachea were of normal capacity, their lumen was patent. The esophageal lumen was of normal size; the mucosa was pale.

### Microscopic evaluation

**Fig. 2:**
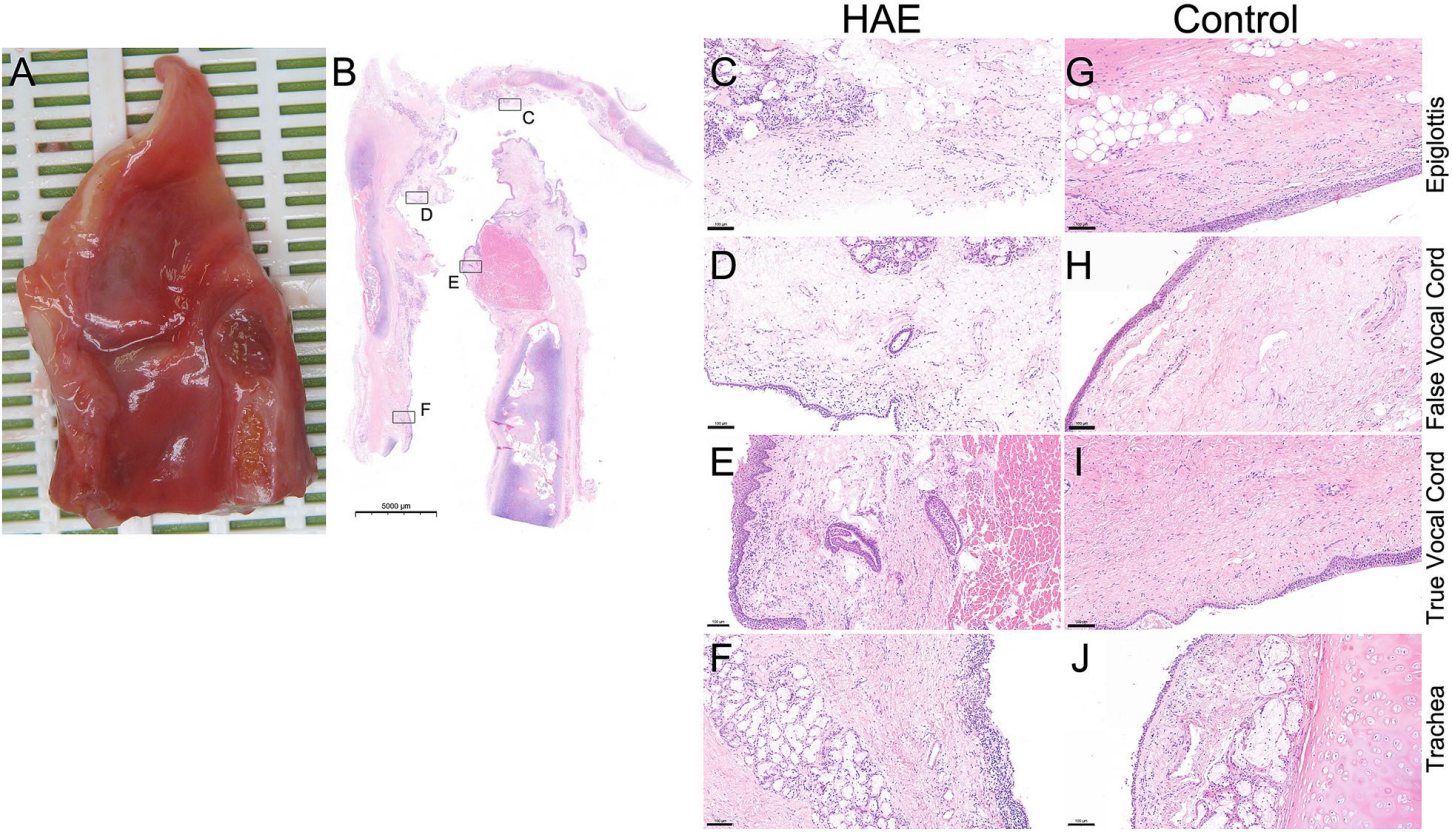
Histological comparison of hematoxylin–eosin stained sections from HAE patient and control. **A** Macrophoto of a median-sagittal opened HAE patient’s sample. **B** H&E staining of the median-sagittal section. **C**–**F** Enlarged images from 4 locations of panel B photo. **G**–**J** Corresponding images to (**C**–**F**) from the control sample. Scale bar: panel B 5000 μm, panels (**C**–**J**) 100 μm

To descript the exact location of the edema in the HAE patient, which directly caused suffocation, we applied routine histology staining. In HAE patient, hematoxylin–eosin staining revealed an extended edema at the epiglottis and the false vocal cord, whereas it was less pronounced at the areas of the true vocal cord and the trachea. Epithelial surface was substantially eroded at the epiglottic area. No similar edematous area or epithelial injury could be observed in the sample of the control patient (Fig. 2). The microscopy pictures of the edematous areas correlated with the macroscopic findings.

### Comparison of HAE and control samples by immunohistochemistry

In our previous studies, we found elevated white blood cell count in HAE patients compared to healthy controls [12]. Based on CD3 and CD11b staining, we found stronger T cell and myeloid cell infiltration in the mucosa/submucosa region of the HAE patient than in the control. In contrast, no B cell infiltration was observed in either cases (data not shown).

**Fig. 3:**
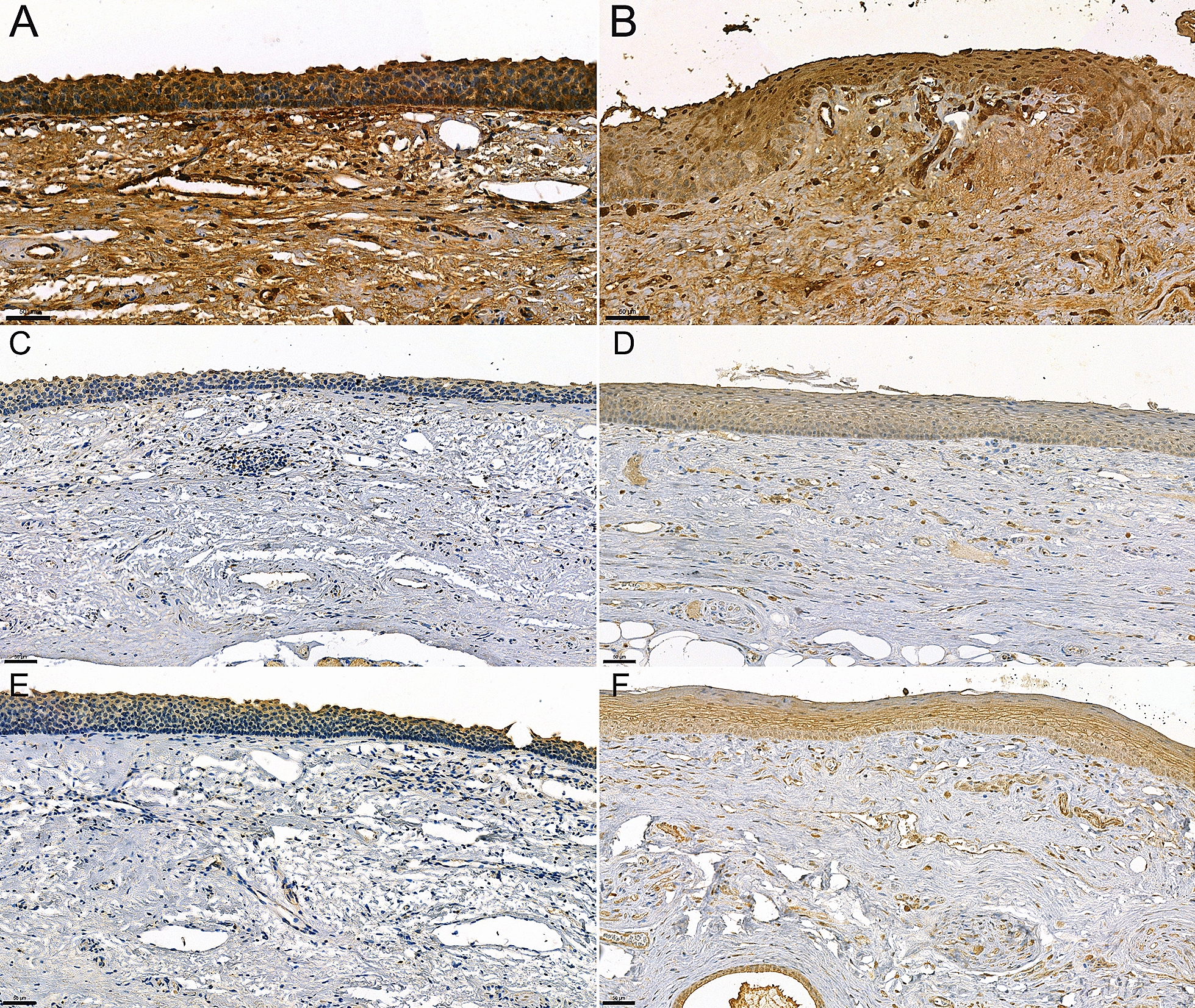
Immunohistochemical staining of C1-Inhibitor and bradykinin receptors. HAE (**A**) and control (**B**) epiglottis samples were incubated with affinity purified rabbit anti-human C1-Inhibitor antibody followed by peroxidase conjugated goat anti-rabbit. The immune reaction was developed by DAB and counterstained with hematoxylin. HAE patient’s (**C**, **E**) and control’s (**D**, **F**) epiglottis samples were incubated with rabbit anti-BDKRB1 antibody (**C**, **D**) or anti-BDKRB2 antibody (**E**, **F**) followed by a peroxidase conjugated goat anti-rabbit secondary antibody. The immune reaction was developed by DAB and counterstained with hematoxylin. Scale bar: 50 μm

Next, we asked what the histological pattern of C1-INH was in HAE patient, and whether this pattern or the intensity of C1-INH staining was different from that of the control. Since the HAE patient had a C1-INH-HAE type 2 disease with normal C1-INH concentration but with decreased C1-INH function, we expected moderate difference in C1-INH concentration compared to the control patient. Indeed, a weaker overall C1-INH signal was detected in the control as opposed to the HAE patient (Fig. 3A, B).

The pattern of C1-INH staining was similar in the HAE- and control patient, i.e. interstitial space, vessel lumen, epithelial cells (including glandular tissues), endothelial cells (ECs), muscle cells and leukocytes were intensively stained, whereas the examined cartilage tissues were negative at all four locations (epiglottis, true vocal cord, false vocal cord, trachea) (data not shown).

Bradykinin receptors have great importance in HAE since they mediate the edematogenic response of bradykinin towards endothelial cells. Both receptors (BDKRB1, BDKRB2) were expressed in epithelial cells, leukocytes and ECs (Fig. 3C–F). Although a slightly lower expression of both BDKRBs was observed in HAE patient than in the control, the pattern of expression was similar in both cases.

**Fig. 4:**
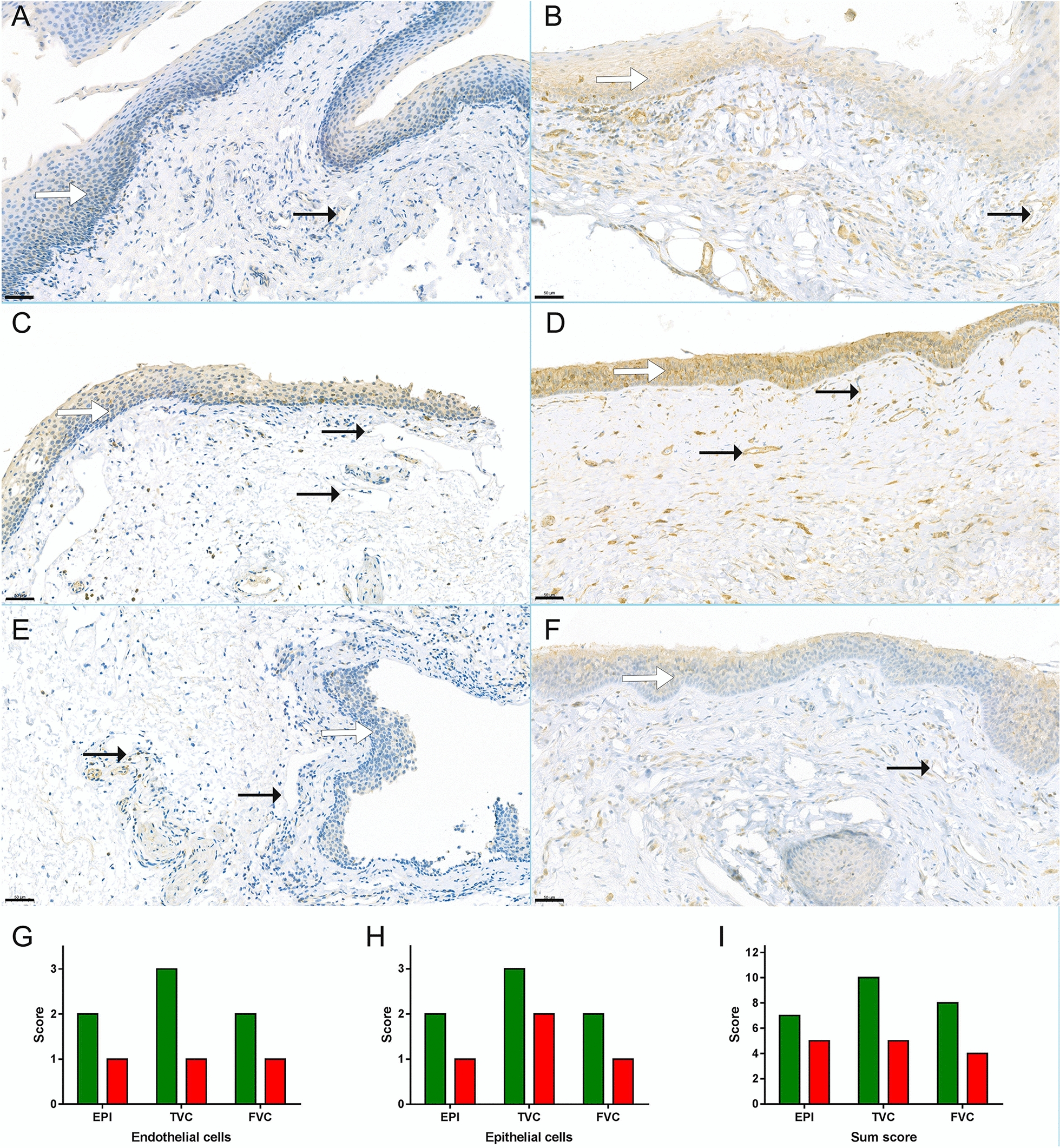
Expression of PAR1 in HAE patient and control. Samples were incubated with rabbit anti-PAR1 antibody followed by peroxidase conjugated goat anti-rabbit secondary antibody. The immune reaction was developed by DAB and counterstained with hematoxylin. HAE patient’s (**A**, **C**, **E**) and control’s (**B**, **D**, **F**) epiglottis (**A**, **B**), true vocal cord (**C**, **D**) and false vocal cord (**E**, **F**) regions are shown. Black arrows indicate endothelial cells and white arrows indicate epithelial cells. Scale bar: 50 μm. Expression pattern of PAR1 in different anatomical locations is compared as staining intensity scores. Green bars represent the control subject’s values, red bars represent the HAE patient’s values. Sum scores (**I**) were calculated by the summation of epithelial cells (**G**), endothelial cells (**H**), leukocytes and interstitial space staining intensity scores (individually ranged from 0 to 3). (EPI: epiglottis, TVC: true vocal cord, FVC: false vocal cord.)

We recently showed that several activated plasma serine proteases, which can be inhibited by C1-INH, induce elevated EC permeability in vitro, predominantly via the PAR1 signaling pathway. We observed significantly lower expression of PAR1 in HAE patient than in the control sample. Interestingly, this difference was not restricted to the ECs and epithelial cells, but muscle cells were also included (Fig. 4A–I). We did not find similar straight-forward difference in the expression of other two PARs: PAR2 and PAR4 (data not shown).

## Discussion

This is the first immunohistochemical comparison of the laryngeal tissue of a patient with C1-INH-HAE who died from suffocation caused by upper airway angioedema with a control patient who died from other condition without any signs of angioedema. We found strong C1-INH staining in both patients—i.e., in the C1-INH-HAE and control patient-, besides a slightly decreased BDKRB1 and BDKRB2 expression and more intensive T cell/monocyte infiltration in the C1-INH-HAE patient. Interestingly, PAR1 expression was significantly lower in the C1-INH-HAE patient, which may reflect to receptor consumption due to PAR1 activation.

The major limitation of our study is that only a single HAE patient’s data have been involved, so every observation of ours should be considered with special care. Although obtaining in vivo samples from angioedematous tissues raises ethical issues, in particular, the biopsy (a known trigger factor of AE attacks) may further worsen the patient’s condition—especially in the case of submucosal edema formation [13], our observations urge these in vivo biopsy studies to clarify the local role of PAR1, BDKRBs and active serine proteases. Skin biopsy studies during HAE attacks would be even more important because a submucosal tissue biopsy is hardly achievable during attacks, and because of the almost negligible chance for obtaining *post mortem* specimen from the larynx of a C1-INH-HAE patient who succumbs to suffocation from laryngeal edema. Specifically, UAE is rare in C1-INH-HAE—occurring in approximately 0.9% of all AE attacks—and the proportion of patients who have ever experienced UAE during their lifetime is 50% (Bork et al.[4] and our unpublished data). Furthermore, nowadays, patients are sufficiently informed about the risk of UAE, as well as they are adequately trained and supplied with emergency treatments [14]. Consequently, the risk of fatality from UAE is extremely low.

Considering the macroscopic picture of laryngeal edema, we found that submucosal edema localized mainly at the epiglottic and false vocal fold areas, and the true vocal folds were less involved. These findings are similar to the previous observations [9]. Hematoxylin–eosin staining of the laryngeal tissue confirmed the macroscopic observations.

We demonstrated moderate leukocyte infiltration in the C1-INH-HAE patient’s laryngeal tissue. We previously described that the level of several homing chemokines was elevated during HAE attacks [12], which may explain the leukocyte infiltration.

Our patient with type 2 C1-INH-HAE had an elevated C1-INH plasma concentration. In line with this, we demonstrated a slightly more intense C1-INH staining in the C1-INH-HAE patient than in the control patient. The staining pattern showed that endothelial cells, epithelial cells, muscle cells, leukocytes and interstitial space all contain a significant amount of C1-INH, which suggests that—besides the liver as the primary source of C1-INH production—the other tissues/cells are also able to produce and/or store C1-INH. These data are in line with the previous findings [15–18], which demonstrated that fibroblasts, monocytes and endothelial cells can synthesize C1-INH.

We found an interesting and surprising staining pattern regarding the bradykinin receptors. The literature is controversial in this field: BDKRB2 was considered as the constitutive and BDKRB1 as the inducible bradykinin receptor [19], although in meta-data bases both bradykinin receptors are widely expressed in different tissues (https://www.proteinatlas.org/ENSG00000100739-BDKRB1, https://www.proteinatlas.org/ENSG00000168398-BDKRB2). In our cases, both bradykinin receptors were expressed in the C1-INH-HAE patient and in the control patient, too. The expression of both bradykinin receptors was slightly lower in C1-INH-HAE patient than in the control patient. This suggests that both bradykinin receptors were activated during the HAE attack of our patient.

Our most important and novel observation was that the PAR1 expression was strongly reduced in the C1-INH-HAE patient compared to the control patient. In our previous in vitro study, we showed that PAR1 activation by thrombin, MASP-1 [7] and other plasma serine proteases controlled by C1-INH [8] leads to endothelial cell hyperpermeability. During this PAR1 activation, the receptor is cleaved, and the immunohistochemical reactivity decreases compared to the intact form. These previous findings of ours harmonize with the reduced expression of PAR1 in the C1-INH-HAE patient’s laryngeal edematous tissue, which suggests an important role of MASP-1 and other plasma serine proteases in the pathogenesis of angioedema. Our hypothesis regarding PAR1-cleaving serine protease activation during HAE attacks is further supported by several previous studies, where elevated activation of the coagulation-, complement- and fibrinolytic-system was found [20–22]. Based on our observation, both PAR1 and its activator serine proteases (all of which are current therapeutic targets in other diseases) may be considered as new therapeutic targets in HAE if further studies involving tissue biopsy can confirm our results.

Finally, if our observations, derived from the sample of a type 2 C1-INH-HAE patient, are validated, they may well be applicable for the pathomechanism of type 1 C1-INH-HAE and probably (with some restrictions) for that of other BK mediated angioedemas.

In conclusion, studying the molecular and cellular mechanisms in human angioedematous tissues is a necessary future target besides blood plasma analysis. On the one hand, our unique case and novel results confirmed those observed in plasma (e.g., regarding data on C1-INH), on the other hand, it suggested new information about the pathomechanism HAE attack, which seems to be much more complex than we had thought before.

## Data Availability

Restricted amount of embedded unstained histology blocks is available upon contact Henriette Farkas.

## Abbreviations

AE: Angioedema
AgC1-INH: C1-inhibitor concentration (antigen)
Anti-C1-INH Ab: Autoantibodies against C1-inhibitor
BDKRB1/2: Bradykinin B1/B2 receptor
C1q, C3, C4: Complement system components
C1-INH: C1-inhibitor
C1-INH-HAE: Hereditary angioedema with C1-inhibitor deficiency
CH50: Total complement activity
fC1-INH: C1-inhibitor function
HAE: Hereditary angioedema
MASP-1: Mannose-binding lectin associated serine protease 1
pdC1-INH concentrate: Plasma-derived C1-inhibitor concentrate
PAR: Protease activated receptor
